# Non-Apoptotic Role of Apoptotic Caspases in the *Drosophila* Nervous System

**DOI:** 10.3389/fcell.2022.839358

**Published:** 2022-02-09

**Authors:** Sarah Colon-Plaza, Tin Tin Su

**Affiliations:** Department of Molecular, Cellular and Developmental Biology, University of Colorado, Boulder, CO, United States

**Keywords:** Drosophila, apoptosis, caspase, central nervous system, peripheral nervous system

## Abstract

An increasing number of studies demonstrate that cells can activate apoptotic caspases but not die and, instead, display profound changes in cellular structure and function. In this minireview, we will discuss observations in the nervous system of *Drosophila melanogaster* that illustrate non-apoptotic roles of apoptotic caspases. We will preface these examples with similar observations in other experimental systems and end with a discussion of how apoptotic caspase activity might be constrained to provide non-lethal functions without killing the cell.

## Introduction

Caspases are proteases that exist as multi-member families in metazoan numbering, for example, twelve in human and seven in *Drosophila melanogaster* (Denton et al., 2013; Kesavardhana et al., 2020). Caspase-like proteins have been identified also in fungi and plants (Minina et al., 2017). Some members of the caspase family, for example human caspases 1 and 4 and *Drosophila* Death related ced-3/Nedd2-like caspase (Dredd), have dedicated roles in non-apoptotic processes such as inflammation and immunity. Other members, for example human caspases 3 and 9 and *Drosophila* Death regulator Nedd2-like caspase (Dronc) and Death related ICE-like caspase (Drice), are essential for apoptosis and will be referred to as “apoptotic caspases.” An increasing body of literature, however, documents non-apoptotic functions of apoptotic caspases, in altering cell identity, sub-cellular remodeling, and production of extra-cellular signals, to name a few. Such apoptotic caspase-driven alterations in cellular structure and function are found in different cell types and across diverse organisms. Here, we will use examples from the *Drosophila* nervous system to illustrate multiple non-apoptotic roles of apoptotic caspases. We will spring-board off excellent reviews on the subject [for example, (Yamaguchi and Miura, 2015; Melzer and Broemer, 2016; Nakajima and Kuranaga, 2017)], to focus on primary papers published since the publications of these reviews and provide an up-to-date summary.

## Activation of Apoptotic Caspases

**FIGURE 1:**
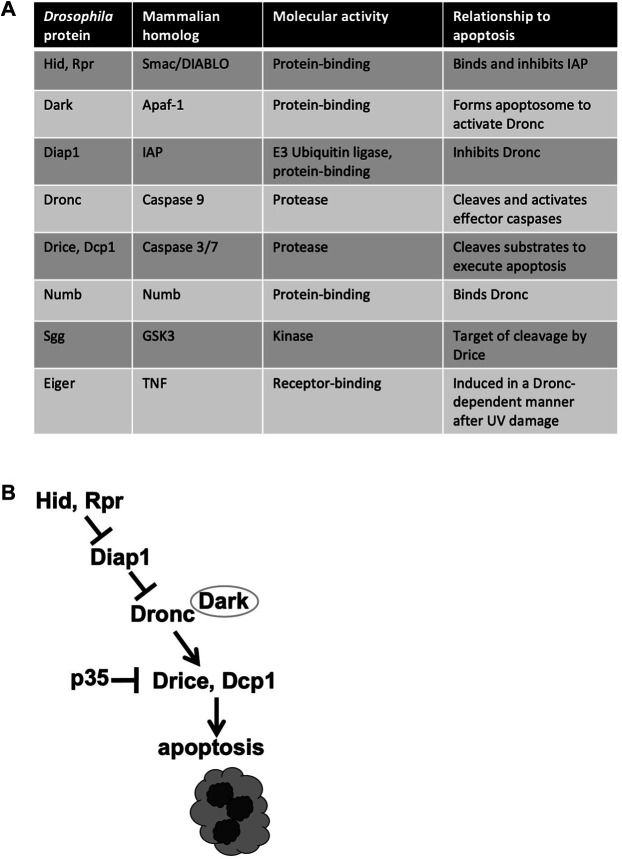
Apoptotic and related proteins discussed in this review. **(A)** The names of *Drosophila* and mammalian homologs, along with the known functions. **(B)** The apoptotic signaling pathway. Only the components discussed in this minireview are shown.

Caspases are cysteine proteases, that is, they require a cysteine in the active site for activity. Caspases are produced as inactive proenzymes that become activated upon proteolytic cleavage. Activation of apoptotic caspases occurs in a cascade that begins with internal or external death stimuli such as DNA damage. In *Drosophila*, exposure to ionizing radiation (IR) leads to transcriptional activation of pro-apoptotic proteins Head Involution Defective (Hid) and Reaper (Rpr) (Brodsky et al., 2004; Wichmann et al., 2006) (Figure 1). These proteins antagonize Death-associated inhibitor of apoptosis 1 (Diap1) to result in the release and activation by cleavage of Dronc at the apoptosome, a multi-protein structure formed by Death-associated APAF1-related killer (Dark) [reviewed in White et al. (2017)]. Dronc is an apical/initiator caspase that in turn cleaves to activate effector/executioner caspases Drice and Death caspase 1 (Dcp1). Genetic analysis demonstrates that Dronc and Drice are required for DNA damage-induced apoptosis while Dcp1 finetunes this process to accelerate the onset of apoptosis (Florentin and Arama, 2012). Viral caspase inhibitor p35 inhibits effector caspase activity but not initiator caspase activity (Meier et al., 2000; Yoo et al., 2002), and has been used to distinguish the requirements for these two classes of apoptotic caspases.

## *Drosophila* Nervous Systems

The Central and Peripheral Nervous Systems (CNS and PNS) of a newly hatched *Drosophila* larva are generated from the embryonic neuroectoderm through sequential cell fate specification events (Singhania and Grueber, 2014; Crews, 2019). Briefly, clusters of cells in the embryonic ectoderm first acquire neuronal competence through the expression of pro-neural transcription factors. Neurogenic factors such as Notch and Delta then specify a single neuronal precursor cell within each cluster. Neuronal precursor and selector genes then allow neuronal precursors to differentiate into neuronal progenitor cells. Neuronal progenitors are known by different names depending on whether they are in the CNS or PNS and what types of neurons they produce. For example, CNS progenitors are called neuroblasts while PNS progenitors that produce extrasensory organs are called Sensory Organ Precursors (SOPs). Neuronal progenitor cells undergo stereotypical asymmetric cell divisions to produce two different daughter cells. Ensuring different identities in the cellular progeny requires Numb protein, which is partitioned to just one of the two products of cell division (Rhyu et al., 1994). Numb is a membrane-bound protein with no known biochemical activity that functions through protein-protein interactions. In the progeny of a neuroblast that received Numb, for example, it inhibits Notch signaling to allow differentiation while the other progeny retains the neuroblast identity. Thus, Numb helps limit the number of progenitor cells to prevent hyperproliferation in the CNS. Neuronal cell number is further limited by Programmed Cell Death, an integral part of CNS development that kills by apoptosis approximately one third of CNS cells that are ever born (Crews, 2019). In this context of massive apoptosis, apoptotic caspases provide activities that do not result in cell death as described next.

### Changing Cellular Identity

Metazoan development requires cells to go through several identity changes as they transition from progenitor to a fully differentiated state. Given that the genome remains unchanged, developmental changes in cellular identity must occur through interconnected changes in the transcriptome and in the proteome. As proteases, apoptotic caspases can alter the proteome as seen in numerous examples. Transcription factor Paired Box 7 (Pax7) maintains muscle stem cell identify but its cleavage by apoptotic caspases allows differentiation into myoblasts, a step inhibited by a caspase resistant form of Pax7 (Dick et al., 2015). Caspase 3 null mutant (caspase 3^−/−^) mice that survive to near-birth stages show reduced skeletal muscle mass. Isolated myoblasts from such mice failed to differentiate into myotubes in culture, indicating a requirement for caspase activity also in a late step in muscle differentiation (Fernando et al., 2002). This requirement may be through Mammalian Sterile Twenty-like kinase (MST1) because it includes caspase consensus sites and a shortened MST1 that mimics the cleaved product rescued differentiation in *caspase 3* null mutant cells (Fernando et al., 2002; Murray et al., 2008). Additional instances of cellular differentiation, in either normal development or regeneration, show a requirement for apoptotic caspase activity without accompanying evidence for apoptosis [for example, (Fernando et al., 2002; Fernando et al., 2005; Koto et al., 2009),]. Caspase substrates that must be cleaved to allow differentiation include pluripotent factors such as Nanog Homeobox (Nanog) in mouse embryonic stem cells (Fujita et al., 2008) and Abnormal Cell Lineage 29 (Lin-29) during *C. elegans* larval development (Rougvie and Moss, 2013; Weaver et al., 2014). Likewise, apoptotic caspases contribute to two instances of cellular identity change in the *Drosophila* nervous system as seen in the following examples.

**FIGURE 2:**
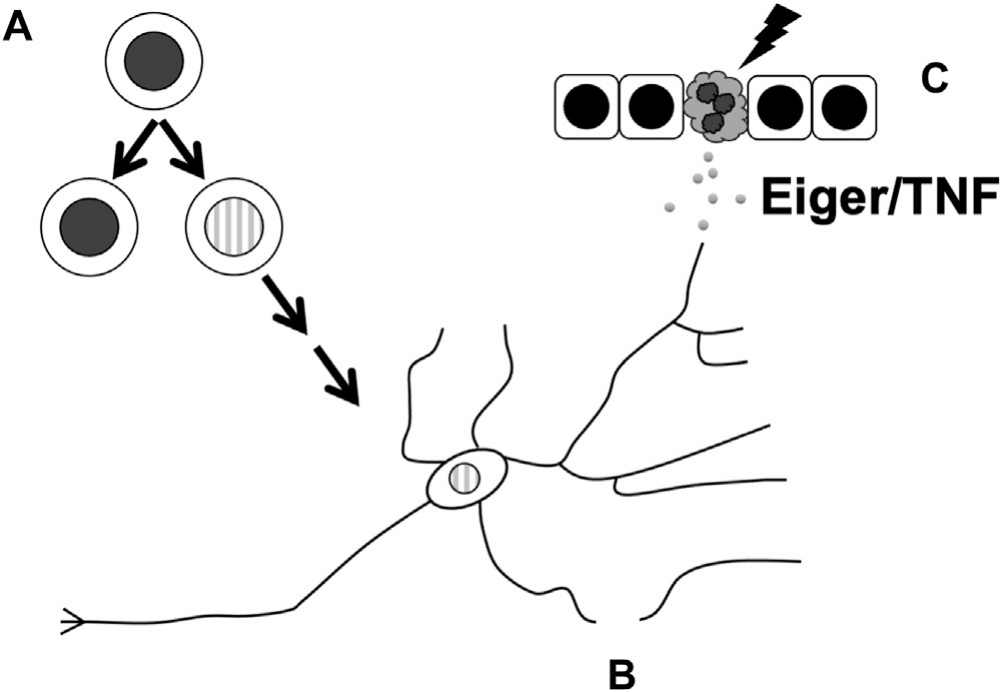
Cellular processes in the *Drosophila* nervous system that are affected by non-apoptotic activities of apoptotic caspases. These include **(A)** limiting neuronal progenitor cell number by ensuring the fidelity of cell fate specification in the PNS or asymmetric divisions in the CNS, **(B)** subcellular remodeling during the pruning of dendrites, and **(C)** facilitating the release of Eiger/TNF from UV-damaged epidermis to result in sensitization of nociceptor neurons.

In the first example, apical caspase Dronc binds Numb to prevent the hyperproliferation of neuroblasts (Ouyang et al., 2011) (Figure 2A). As described above, partitioning of Numb to just one of two cells produced during an asymmetric neuroblast division allows the said recipient to differentiate. Without Numb, both daughter cells retain their progenitor status, leading to ectopic neuroblast formation (ENF) and hyperplasia. A yeast 2-hyrid screen identified Dronc as an interactor of Numb, and Dronc overexpression rescued the ENF phenotype caused by the expression of a dominant-negative *numb* mutant. This rescue required the presence of wild type Numb, suggesting that the rescue occurs through sequestration of mutant Numb to allow the wildtype Numb to work. The rescue did not require effector caspase activity or the catalytic activity of Dronc, suggesting that Dronc acts in a non-apoptotic manner. Full length Dronc binds Numb as well as cleaved active Dronc, suggesting that any activity provided by Dronc to limit neuroblast number can occur without fear of killing the cell. *dronc* loss-of-function mutants do not have extra neuroblasts but exacerbate the milder ENF phenotype of weak dominant negative *numb* mutants. Therefore, Dronc appears to provide an important but redundant function to limit neuroblast number.

Apoptotic caspase activity also limits the number of SOPs, progenitor cells in the PNS (Figure 2A). A subset of SOPs produce external sensory organs called macrochaetes that are readily visible on the back of the adult fly, specifically, in a triangular region called the scutellum. Mutations in *dark* or *dronc* as well as inhibition of caspase activity with a dominant-negative Dronc (*Dronc* ^*DN*^) or p35 in the scutellum produced extra SOPs and extra macrochaetes. One possible explanation for this phenotype is that caspases normally reduce SOP cell numbers through apoptosis. But this possibility was rendered unlikely by the absence of TUNEL-positive cells in the pro-neural clusters that produce the SOPs. Instead, caspases may suppress Wingless (Wg, *Drosophila* Wnt1) signaling to limit the SOP number; a mutant allele of *wg* suppressed the extra macrochaetes phenotype that results from *Dronc* ^*DN*^ while null mutants in Wg inhibitor *shaggy* (*sgg*, encoding *Drosophila* GSK3 kinase) enhanced this phenotype. Sgg contains two caspase 3/7 consensus sites. Expression of Sgg with caspase consensus sites mutated produced extra SOPs and macrochaetes, leading to the model that caspases cleave to activate Sgg, which then opposes Wg to limit the SOP number (Kanuka et al., 2005). As the non-cleavable Sgg produced a phenotype in the presence of endogenous wild type Sgg, the former was proposed to act as a dominant negative against the latter. How might caspase activity finetune SOP number without killing the cells? Additional studies provide evidence that *Drosophila* I-kappaB kinase (IKK)-like kinase regulates the turnover of Diap1 to temper caspase activity and enables it to provide a developmental role without inducing cell death (Kuranaga et al., 2006; Koto et al., 2009).

While these studies provide compelling evidence for a non-apoptotic role of apoptotic caspases in *Drosophila* PNS, the data also suggest complexities. For instance, non-cleavable Sgg was less effective than inhibition of Dark or Dronc in producing extra macrochaetes, leading the authors to suggest additional substrates besides Sgg at play (Kanuka et al., 2005). A more recent paper addressed this possibility directly by using CRISPR to replace endogenous Sgg with non-cleavable Sgg (Wang and Baker, 2019). The resulting flies, surprisingly, show normal SOP and macrochaete numbers. These results can be reconciled if non-cleavable Sgg acts not by opposing endogenous Sgg but another protein that is the *bona fide* caspase substrate in controlling SOP number. In other words, while the data support the conclusion that apoptotic caspases play a non-apoptotic role in controlling SOP number, we may not yet know their relevant substrates in this process. The newer study identified an additional upstream regulator of non-apoptotic caspase function in limiting SOP number. Inhibition with mutations or RNAi of *expanded (ex)*, which encodes for a signal transducer in the Hippo (Hpo) pathway, produced extra macrochaetes as did over-expression of transcription factor Yorki (Yki, *Drosophila* YAP), an inhibitory target of Hpo signaling. The macrochaetes phenotype of *ex* mutants was rescued by reducing *diap1*, a known transcriptional target of Yki (Wang and Baker, 2019). These results suggest that Hpo/Yki tumor suppressor pathway regulates Diap1 transcriptionally in SOP number determination. This mechanism could operate in parallel with the IKK-mediated mechanism that controls Diap1 protein turnover (Kanuka et al., 2005).

### Sub-Cellular Sculpting

During apoptosis execution, caspase activity helps destroy organelles such as the nucleus and the Golgi (Chiu et al., 2002; Al-Ghorbani et al., 2016). Likewise, caspase activity helps eliminate organelles in some instances of cellular remodeling that accompany differentiation. The removal of the nucleus from the lens cells for light transmission and from red blood cells for efficient oxygen transport requires caspases (Wride et al., 1999; Zermati et al., 2001). In *Drosophila*, Drice is needed to reduce the cytoplasm and associated organelles during the final step of sperm differentiation (Arama et al., 2003). Inhibiting effector caspase activity with p35 prevented cytoplasm elimination and produced sterile males, illustrating the importance of caspase activity in sperm development. The following examples from two sets of neurons illustrate that subcellular sculpting by apoptotic caspases occurs also in the *Drosophila* PNS (Figure 2B).

Larval PNS neurons prune their dendrites as the larva metamorphoses into an adult. A genetic screen identified ubcD1, an E2 ubiquitin-conjugating enzyme, as a requirement for pruning in C4da neurons (Kuo et al., 2006). A relevant target of this degradation system appears to be Diap1; a *diap1* mutant that was an inefficient substrate for degradation acted as a dominant negative to inhibit pruning. *dronc* mutants show pruning defects, suggesting that degradation of Diap1 allows Dronc activation and pruning. How is cell death avoided in such a scenario? An antibody against cleaved human caspase 3 shows signal only in dendrites and not in axons and only during the period of dendrite severing. Thus, Diap1 may be degraded locally to produce localized caspase activity that affects localized subcellular remodeling without endangering the cell. Interestingly, expression of p35, which inhibits effector caspases, did not affect pruning, suggesting that apical caspase activity but not effector caspase activity promotes pruning in C4da neurons. In ddaC neurons, a genetically encoded caspase activity reporter shows spatially-restricted caspase activation in dendrites undergoing pruning (Williams et al., 2006). Overexpression of Diap1, Dronc^DN^ or p35 inhibited pruning in ddaC neurons suggesting that both apical and effector caspase activities are needed to prune these neurons. Global caspase activation by overexpression of *hid* resulted in ddaC cell death (Williams et al., 2006), providing a contrast to localized caspase activation that allows pruning without cell death.

A more recent study shows that apoptotic caspases play a non-apoptotic role in a phenomenon called neuroprotection that occurs during axonal regeneration (Chen et al., 2016). Briefly, severing of an axon (axotomy) in *Drosophila* sensory or motor neurons results in neuroprotection (NP) that acts through Jun N-terminal kinase (JNK) and Mitogen-Activated Protein kinase (MAPK). NP can be visualized as preservation of severed dendrites following axotomy and is proposed to buy neurons time to maintain structure and function through the period of axon regeneration. RNAi against *dronc* or mutations in *drice* increased the fraction of dendrites protected in ddaE neurons, suggesting that these caspases normally contribute to the removal of severed dendrites, much like in pruning, and must be overcome for NP. The authors then tested the hypothesis that uncontrolled NP might interfere with subsequent axon regeneration. Indeed, neurons with *dronc* RNAi showed reduced regeneration, leading to the conclusion that Dronc activity promotes axon regeneration by limiting NP.

Neuronal pruning by localized apoptotic caspase activity in *Drosophila* has parallels in mice where proteasome activity and localized expression of an IAP protein keep the activity of caspases 3 and 9 away from the cell body and restricted to axons to be pruned (Cusack et al., 2013).

### Sub-Cellular House-Cleaning

Fungi and plants encode metacaspases, which are proteases that share similar tertiary structure and an active-site cystine with caspases but cleave after Arg or Lys instead of Asp [reviewed in (Minina et al., 2017)]. Budding yeast metacaspase YCA1 is dispensable for cell death (Hauptmann et al., 2006; Kang et al., 2008; Palermo et al., 2013) but *yca1* mutants show elevated stress response proteins, cytoplasmic protein aggregates and reduced replicative lifespan (Lee et al., 2010; Hill et al., 2014). These data suggest a non-lethal role for a metacaspase in removing harmful protein aggregates from the cytoplasm. Paradoxically, apoptotic caspases may provide an opposite function, to clutter rather than to clean, in Alzheimer’s disease (AD) where microtubule-binding protein Tau forms aggregates called neurofibrillary tangles (NFT) (Medeiros et al., 2011). Several lines of data suggest that cleavage of Tau by apoptotic caspases leads to NFT. Tau bears a caspase 3-consensus site and the caspase-cleaved form of Tau as well as cleaved (active) caspase 9 are detected with specific antibodies in neurons from AD patients but not non-AD controls (Rohn et al., 2002; Gamblin et al., 2003). In mouse disease models, activation of effector caspases precedes NFT formation and caspase-cleaved Tau is sufficient to promote NFT formation in wild type brains (De Calignon et al., 2010). Finally, caspase-cleaved Tau forms fibrils more rapidly than full length Tau *in vitro* (Gamblin et al., 2003). Collectively, these data lead to the model that apoptosis caspase activity that is not immediately followed by cell death results in Tau NFTs and disease.

A *Drosophila* model of AD demonstrates a non-lethal role of apical caspase Dronc in Tau cleavage and enabled an investigation of the observed correlation between circadian dysregulation and susceptibility to neurodegeneration (Means et al., 2015). Disruption of circadian rhythm by RNAi-mediated knock-down of circadian kinase encoded by *double time* (*dbt*) or its genetic interactor *spaghetti* in *Drosophila* clock neurons led to Dronc activation as detected with an antibody against the cleaved form. Dronc activation in these experiments was observed only during daytime or after light exposure during the night, could spread to nearby non-clock cells, and increased with fly age. Expression of a dominant-negative *dbt* in the *Drosophila* eye led to Dronc activation in this tissue, cleavage of co-expressed human Tau in a Dronc-dependent manner, and neurodegeneration phenotypes. These results in *Drosophila* parallel the relationship between apoptotic caspase activity, Tau and neurodegenerative disease observed in mammals as described in the preceding paragraph. Furthermore, *Drosophila* studies add to this picture by identifying Dronc activation as a possible mechanism that links circadian dysregulation with susceptibility to neurodegeneration.

### Signaling to Other Cells

Studies in mice and *Drosophila* show that dying cells send mitogenic signals that promote tissue homeostasis. In *Drosophila* larval wing imaginal discs, Dronc promotes Apoptosis-induced Proliferation (AiP) through different mechanisms. First, Dronc acts together with JNK to produce Wg that acts as a secreted mitogen (Perez-Garijo et al., 2004; Ryoo et al., 2004; Kondo et al., 2006). Second, Dronc activation leads to the elevation of extracellular Reactive Oxygen Species, which recruits macrophages that secrete Eiger/Tumor Necrosis Factor (TNF) to activate JNK and sustain mitogenic signaling (Fogarty et al., 2016; Khan et al., 2017). The role of Dronc in AiP is distinct from its role in activating effector caspases for apoptosis because AiP still occurs when the effector caspases are inhibited. In mice, effector caspases 3/7 cleave and activate Calcium-independent phospholipase A2 to result in the generation and release of prostaglandin E, a known promoter of cell proliferation (Li et al., 2010; Huang et al., 2011). Thus, apoptotic caspase activity produces non-cell autonomous mitogenic signaling in both *Drosophila* and mammals, albeit through different downstream targets.

A parallel story has emerged recently in *Drosophila* thermal nociception (sensing painful heat) where Dronc and Eiger function in a non-apoptotic manner to promote sensitization to pain (Jo et al., 2017) (Figure 2C). The exposure of *Drosophila* larval *epidermis* to UV results in both apoptosis and allodynia (extreme sensitivity to pain). The latter outcome can be observed as an adverse response to thermal stimuli that would not elicit a response in non-irradiated animals. Dronc is required for both UV-induced apoptosis and thermal allodynia while effector caspases Drice and Dcp1 are required only for apoptosis, suggesting that Dronc plays a non-apoptotic role to induce allodynia. This idea is supported by the finding that doses of UV that are too low to induce apoptosis still induced thermal allodynia in a Dronc-dependent manner. Dronc, it was found, acts in the *epidermis* to produce Eiger/TNF that then signals through TNF Receptor (TNFR) on sensory neurons to induce thermal allodynia. Induction of Dronc in the *epidermis*, even when effector caspases are inhibited to prevent Dronc-induced apoptosis, results in thermal allodynia in an Eiger-dependent manner without UV exposure, further lending evidence to a non-apoptotic role for this apoptotic caspase.

## Remaining Questions

Given that activation of apoptotic caspases can lead to apoptosis, how do cells restrict this activity for other purposes without being killed? Two mechanisms emerge from the examples described here: keeping caspase activity too low for apoptosis or restricting it spatially. For both mechanisms, regulatory inputs appear to integrate at the level of Diap1, an E3 ubiquitin-ligase that inhibits Dronc *via* mono- or poly-ubiquitination (Lee et al., 2011; Kamber Kaya et al., 2017). For the first mechanism, IKK-dependent regulation of Diap1 degradation and Hpo/Yki-dependent regulation of Diap1 transcription could keep caspase activity below the threshold for apoptosis yet sufficient to regulate SOP number. For the second mechanism, spatial restriction of Diap1 degradation in C4da neurons may be what allows localized caspase activation and dendrite pruning without killing the cell. Other mechanisms besides regulation of Diap1 likely exist but a comprehensive identification of such mechanisms would require the ability to identify cells that activated apoptotic caspases but did not die. Two recently-described biosensors are helping in this regard. Caspase Tracker and CasExpress rely on caspase-mediated recombination events that result in permanent GFP expression, marking cells with past apoptotic caspases activity as well as their clonal descendants (Tang et al., 2015; Ding et al., 2016). These reporters reveal many cell types, including those in the neuronal lineage, that activate apoptotic effector caspases but do not die during *Drosophila* development. An RNAi screen using a version of the CasExpress reporter identified genes that altered the number of living cells with past caspase activity (Sun et al., 2020). Some of these genes act downstream of or in parallel to caspase activation to decide whether a cell that activated apoptotic caspases will survive or not. One of these genes encodes a homolog of human CDKN1A-interacting zinc finger protein 1 (CIZ1); how CIZ1 functions to preserve cells that activated caspases remains to be determined. It would be interesting to see if CIZ1 or other regulators of cell survival after caspase activation identified using the CasExpress reporter play a role in the nervous system.
